# Supplementary material for: Comparative genomics provides new insights into the diversity, physiology, and sexuality of the only industrially exploited tremellomycete: Phaffia rhodozyma
Source: BMC Genomics. 2016 Nov 9;17:901. doi: 10.1186/s12864-016-3244-7 (PMC5103461; doi:10.1186/s12864-016-3244-7)
Supplement: Additional file 6: — List of orphan genes with links to PFAM (related to Additional file 1: Table S1). (ZIP 1428 kb) [file 12864_2016_3244_MOESM6_ESM.zip › PFAM_UNIQUE/G00252_P.html]

#                                                                            --- full sequence --- -------------- this domain -------------   hmm coord   ali coord   env coord
  
# target name        accession   tlen query name           accession   qlen   E-value  score  bias   #  of  c-Evalue  i-Evalue  score  bias  from    to  from    to  from    to  acc description of target
  
#------------------- ---------- ----- -------------------- ---------- ----- --------- ------ ----- --- --- --------- --------- ------ ----- ----- ----- ----- ----- ----- ----- ---- ---------------------
  
G00252\_P             -           1137 MAP7                 PF05672.6    171       1.8    6.6  17.3   1   1      0.02       4.9    5.2  17.3    17   113   623   714   608   722 0.55 - MAP7 (E-MAP-115) family  
G00252\_P             -           1137 UPF0564              PF10595.4    356       7.4    4.0   9.4   1   1     0.069        17    2.9   9.4   267   342   546   701   492   710 0.44 - Uncharacterised protein family UPF0564
